# Multiple delivery strategies of nanocarriers for myocardial ischemia-reperfusion injury: current strategies and future prospective

**DOI:** 10.1080/10717544.2023.2298514

**Published:** 2023-12-26

**Authors:** Shengnan Li, Fengmei Li, Yan Wang, Wenqun Li, Junyong Wu, Xiongbin Hu, Tiantian Tang, Xinyi Liu

**Affiliations:** a Department of Pharmacy, The Second Xiangya Hospital, Central South University, Changsha, China; b Institution of Clinical Pharmacy, Central South University, Changsha, China

**Keywords:** Targeted drug delivery, biomimetic nanocarriers, myocardial ischemia-reperfusion injury, miRNA

## Abstract

Acute myocardial infarction, characterized by high morbidity and mortality, has now become a serious health hazard for human beings. Conventional surgical interventions to restore blood flow can rapidly relieve acute myocardial ischemia, but the ensuing myocardial ischemia-reperfusion injury (MI/RI) and subsequent heart failure have become medical challenges that researchers have been trying to overcome. The pathogenesis of MI/RI involves several mechanisms, including overproduction of reactive oxygen species, abnormal mitochondrial function, calcium overload, and other factors that induce cell death and inflammatory responses. These mechanisms have led to the exploration of antioxidant and inflammation-modulating therapies, as well as the development of myocardial protective factors and stem cell therapies. However, the short half-life, low bioavailability, and lack of targeting of these drugs that modulate these pathological mechanisms, combined with liver and spleen sequestration and continuous washout of blood flow from myocardial sites, severely compromise the expected efficacy of clinical drugs. To address these issues, employing conventional nanocarriers and integrating them with contemporary biomimetic nanocarriers, which rely on passive targeting and active targeting through precise modifications, can effectively prolong the duration of therapeutic agents within the body, enhance their bioavailability, and augment their retention at the injured myocardium. Consequently, these approaches significantly enhance therapeutic effectiveness while minimizing toxic side effects. This article reviews current drug delivery systems used for MI/RI, aiming to offer a fresh perspective on treating this disease.

## 1. Introduction

Cardiovascular diseases (CVDs) are clinically significant diseases with high morbidity and mortality. According to the World Health Organization(WHO), about 17.8 million people died from CVDs each year in 2017, more than three-quarters of them in developing countries, and four-fifths of them died from heart attacks and strokes (Kaptoge et al., 2019). Among CVDs, acute myocardial infarction (AMI) is caused by the occlusion of the epicardial coronary artery, which is one of the most sudden and dangerous diseases. Conventional treatments include percutaneous coronary intervention (PCI) and coronary artery bypass grafting (CABG), which could greatly reduce acute mortality by sparing the infarcted vessel and rescuing the ischemic myocardium in a timely manner but may also paradoxically lead to myocardial ischemia-reperfusion injury (MI/RI). The MI/RI often induces myocardial contractility disorders, arrhythmias, and other adverse consequences (Ibáñez et al., 2015). Long-term myocardial salvage after PCI is thought to be the reason for the rise in patients with heart failure, which went from 18% in 1998 to 30% in 2008 (Jenča et al., 2021). Post-infarction reduction in left ventricular ejection fraction (LVEF) is a prominent contributor to the global prevalence of chronic heart failure (Ibáñez et al., 2015). The underlying mechanisms of MI/RI are complex, including damage from ischemia and hypoxia and subsequent reperfusion injury. Current studies suggest that endothelial dysfunction, immune activation, and inflammatory response are key factors in triggering MI/RI, while reactive oxygen species (ROS), intracellular Ca^2+^ overload, and mitochondrial permeability transition pore (mPTP) opening are crucial molecular mediators. the different modes of cell death and the resulting coronary microvascular dysfunction are the endpoint events of the process (Valikeserlis et al., 2021, Heusch & Gersh, 2017).

Although therapies to reduce ischemic injury have achieved rapid success over the course of several decades, the progress in developing therapies to mitigate reperfusion injury has been unsatisfactory (Heusch & Gersh, 2017). Surgical intervention for ischemic preconditioning is not commonly performed in patients with unpredictable acute myocardial infarction. Ischemic postconditioning, while it has been shown to reduce infarct size in large mammals, does not yield consistent results in large clinical trials (Hahn et al., 2013). Meta-analyses examining distal ischemic preconditioning as a potential approach have consistently demonstrated a statistically significant but modest reduction in myocardial injury among patients with ST-elevation myocardial infarction (STDMI). However, the clinical implications of this reduction are unlikely to be significant (Gong & Wu, 2019, Borracci et al., 2021). A large clinical trial has demonstrated that low-dose anti-inflammatory colchicine has a notable impact in reducing primary endpoint events in individuals who have recently experienced or previously suffered from MI when compared to a placebo (Tardif et al., 2019). However, the clinical trials of cyclosporine A did not demonstrate superior improvement in clinical outcomes when compared to the placebo group (Cung et al., 2015). The limitations of biodistribution, rapid elimination, adverse degradation/biotransformation, short half-life, and poor target specificity were major factors that hindered the effectiveness of drug treatment for MI/RI (Salehi et al., 2020).

Cell therapy, despite being safe, has limited effectiveness, even when administered through intracardiac injection. Human bone marrow stem cells do not undergo transdifferentiation into cardiomyocytes or neovascularization, which are considered the main mechanisms of action. Instead, the primary mechanisms are believed to involve inhibiting apoptosis and fibrosis, improving contractility, and activating regenerative mechanisms through paracrine action (Zhang et al., 2018). Despite intramyocardial delivery, a significant proportion of transplanted cells are lost shortly after the operation, and a gradual decline in cell numbers continues over time (Ottersbach et al., 2018). Therefore, it is clinically important to construct a drug delivery system (DDS) that efficiently delivers therapeutics to the effector sites in the injured myocardium. In terms of drug physicochemical properties, small molecule drugs, nucleic acids, large molecule proteins, and even cells are readily understood within the body and exhibit a brief duration of action (Weng et al., 2022, Tan et al., 2021, Ottersbach et al., 2018). The administered dose does not meet the safety requirements. Carrier encapsulation, particularly when combined with ligand modifications like polyethylene glycol (PEG), can significantly enhance in vivo stability and prolong the circulation time (Li et al., 2018). Another concern is the drug’s off-target effect. Surface functionalization of nanocarriers can enhance their biocompatibility and enable targeted drug delivery, thereby improving safety and efficacy while avoiding immune system clearance (Luk & Zhang, 2015). Moreover, the targeted delivery of engineered nanocarriers can be achieved by exploiting the unique microenvironment of the ischemia-reperfusion site and the external environment, thereby enabling precise control over drug release at the desired location (Wang et al., 2022b, Chen & Li, 2020, Su et al., 2018).

This review presents a summary of polymeric nanoparticles and inorganic nanoparticles that are loaded with therapeutic agents. These nanoparticles have the potential to improve drug delivery and cellular enrichment at sites of myocardial injury. Additionally, they could also improve the inflammatory microenvironment by regulating inflammation, antioxidant effects, and thus promote the repair of ischemic myocardium. Furthermore, we focus on summarizing that the use of ultrasound-targeted microbubbles can effectively enhance stem cell homing and increase gene transfection efficiency, resulting in significant improvements in myocardial function. We next present the studies of multiple exosomes of diverse cellular sources in the field of nucleic acid delivery for the treatment of MI/RI. Biomimetic agents combined with nanocarriers have gained attention for their potential to advance innovation in the field of cardiovascular diseases. These agents possess multiple natural modifications that provide additional biological functions to the carriers. This discussion outlines the role of injectable hydrogels in enhancing myocardial repair at the site of myocardial infarction. This review explores recent advancements in the mentioned systems and discusses the challenges faced by carriers. Relevant data are presented in the table below. The delivery systems for myocardial ischemia-reperfusion injury in recent years are summarized in Table 1.

**Table 1.** The primary drug delivery system for MI/RI.

Vehicle	Aptamer	Therapeutic agent	Model	Intervention time	Effect	Reference
PLGA nanoparticle	/	Cyclosporine A	Mice	Reperfusion after ligation of 30 minutes	Alleviating oxidative stress and improving ventricular remodeling	(Ikeda et al., 2016)
PLGA nanoparticle	/	Cyclosporine A/ pitavastatin	Mice	Reperfusion after ligation of 30 minutes	Enhancing myocardial protection by regulating mPTP opening and inflammation	(Ikeda et al., 2021)
PLGA nanoparticle	/	Alpha lipoic acid (LA)	Mice	Ligation	Reducing ROS damage and restore heart function after myocardial injury	(Xie et al., 2023)
Polymers of PEG and PPS diblock copolymer	/	Ginsenoside Rg3	Rats	30 min ligation followed by reperfusion.	Improving heart function and reducing infarct size	(Li et al., 2020)
PLGA nanoparticle	RPPT/PEG	SiVCAM-1/dexamethasone (DXM	Rats	Ligation for 30 minutes and reperfusion	Inhibiting neutrophil migration and adhesion, Effectively blocking the recruitment of neutrophils, and interrupting the self-amplifying inflammatory cascade	(Hou et al., 2022)
Polydopamine nanoparticle	PEG	/	Mice	Ligation for 30 min followed by reperfusion	Reducing Fe2+ deposition and lipid peroxidation in myocardial I/R injury mouse model	(Zhang et al., 2021c)
Superparamagnetic nanoparticle	carboxymethyl-dextran polymer	VEGF-165	In vitro	/	Superior effects on cell growth and survival and MRI activity remain in MRI acquisitions	(Bietenbeck et al., 2019)
Nanoparticle consists of a Fe_3_O_4_ core and SiO_2_ shell	PEG	CD63-expressing exosomes	Rats/ Rabbits	Ligation	The decrease in infarct size and the improvement in left ventricular ejection fraction and angiogenesis	(Liu et al., 2020)
Mesoporous silica nanoparticle	CD11b	Notoginsenoside R1	Mice	Ligation	Modulating macrophage phenotype and promoting angiogenesis	(Li et al., 2022b)
Mesoporous silica nanoparticle	PPTP	Dexamethasone (Dex)/ RNA (siRAGE)	Rats	Ligation for 30 minutes and reperfusion	Suppression of myocardial fibrosis and apoptosis and restoration of myocardial contractile function	(Lan et al., 2022)
Mesoporous silica nanoparticle	PNNTBA	H_2_S	In vitro	/	Decreased expression of inflammatory factors TNF-α and IL-1β, apoptosis rate, and lactate dehydrogenase activity	(Xia et al., 2022)
Ultrasound targeted microbubble	/	PAd-EGFP/SDF-1α gene	Rats	Ligation	Increasing expression of SDF-1α and the number of BMSCs homing	(Su et al., 2018)
Ultrasound targeted microbubble	PHD2	BMSC	Rats	Ligation	Increasing BMSC survival, decreasing myocardial apoptosis, reducing infarct size, increasing vessel density, and improving cardiac function compared to control vectors	(Sun et al., 2020)
Ultrasound targeted Microbubble	/	PhSDF-1α-NFκB	Rabbits	Ligation	Better recovery of cardiac function, more myocardial perfusion, more neovascularization, smaller infarct size, and thicker infarct layer compared to control	(Yu et al., 2022)
Ultrasound targeted microbubble	/	Ang1 plasmid	Dogs	Ligation	Promoting angiogenesis, reversing LV structure and sympathetic remodeling, and improving LV synchrony after MI	(Cao et al., 2021)
Cationic liposomes with platelet membranes	/	MiR-21	Mice	60 min ligation followed by reperfusion.	Achieve macrophage reprogramming	(Tan et al., 2021)
Platelet membrane chimeric liposomes	DSPE-SeSe-PEG2000	Resolvin D1	Mice	60 min ligation followed by reperfusion.	Enhancing ventricular remodeling and cardiac function in a mouse MI/R model	(Weng et al., 2022)
PLT membrane-coated PLGA nanoparticle	/	Berberin (BBR)	Rats	50 min ligation followed by reperfusion.	Less hepatic uptake in the in vivo pharmacokinetics of rats; reducing the number of myocardial inflammatory and apoptotic cells and cardiac collagen deposition	(Zhu et al., 2023)
PLGA nanoparticle camouflaged by platelet membrane	/	Antagomirs	Rats	30 min ligation followed by reperfusion.	The increasing expression of Nrf2, reducing apoptosis of H9c2 cells, significantly decreasing cytotoxicity of ROS, MDA, and LDH, and enhancing total SOD activity and GPx enzyme activity	(Wang et al., 2022a)
Extracellular matrix (ECM)-derived collagen I hydrogel	/	7-amino acid peptide (7Ap)	Mice	Ligation	Limiting the fibrosis of the left ventricular wall, reducing the thinning of the infarct wall, and significantly improving cardiac performance 2 weeks after MI in mice	(Zhang et al., 2019)
Oligo(poly (ethylene glycol) fumarate) (OPF) hydrogels	/	Graphene oxide (GO) nanomaterials	Rats	Ligation	Maintaining better cardiac function after myocardial infarction compared to injecting a non-conductive polymer	(Zhou et al., 2018)
Injectable hyaluronic acid (HA)- based hydrogel	/	Functionalized MSC aggregates (FMAs)	Rats	Ligation	Improving the microenvironment of MI with reduced expression of inflammatory cytokines and upregulated secretion of angiogenic factors compared to pure hydrogel and hydrogel-coated MSCs	(Lyu et al., 2020)

## 2. Pathogenesis of myocardial ischemia-reperfusion injury

### 2.1. Oxidative stress and inflammation

The concept of ‘oxidative stress’ pertains to a condition wherein there is a disparity between the customary mechanisms of oxidation and scavenging, resulting in the peroxidation of biological macromolecules such as DNA, lipids, and signaling molecules. This, in turn, triggers inflammatory responses and pathways leading to cellular death, thereby inducing a range of biological alterations (Xiang et al., 2021, Du et al., 2023). One of the main sources of ROS is the mitochondria, the center of energy metabolism (Bugger & Pfeil, 2020). The process takes place in a stepwise manner within the mitochondrial electron transport chain (ETC), which comprises four multisubunit complexes (I-IV) that are linked to mobile transporters (coenzyme Q (CoQ) and cytochrome c (Cyt c)). Cardiolipin, a phospholipid situated in the inner mitochondrial membrane, undergoes oxidative damage during reperfusion. This leads to the dissociation of complexes I and III from the supercomplex, resulting in electron leakage from the ETC and the generation of ROS. As such, complexes I and III are the primary sources of ROS during MI/RI. Furthermore, an excessive amount of Ca^2+^ within the mitochondria can lead to the generation of ROS through the tricarboxylic acid cycle (TCA), resulting in a reduction of mitochondrial complex activity and the opening of the mPTP (Hui et al., 2017). The opening of the mPTP has the potential to release ROS from the mitochondria into the cytoplasm, leading to the transmission of localized mitochondrial disturbances to the cardiomyocyte (Zhao et al., 2022). Simultaneously, an overproduction of ROS is frequently concomitant with an inflammatory reaction in the myocardium experiencing ischemia. The aforementioned statement pertains to the liberation of danger-associated molecular patterns (DAMPs) from impaired cardiomyocytes and extracellular matrix. These DAMPs bind to pattern recognition receptors (PRRs) on the surface of inflammatory cells, starting downstream signals from PRRs that activate the NF-κB pathway, mitogen-activated protein kinase pathway, and NLR family pyridine-containing domain protein 3 (NLRP3) inflammasome, leading to the expression of a large number of pro-inflammatory genes. (Ong et al., 2018). At the same time, inflammation of the heart amplifies damage to the myocardium by stimulating the generation of ROS (Cheng et al., 2021).

Macrophages play a crucial role in the pathogenesis of cardiovascular disorders, including but not limited to atherosclerosis, myocardial infarction, and reperfusion injury. The manifestation of macrophage plasticity is indicative of the current stage of inflammation (Algoet et al., 2022). During the initial inflammatory phase, M1 macrophages are activated and secrete inflammatory cytokines such as tumor necrosis factor α (TNF-α), interleukin-1 (IL-1), and interleukin-6 (IL-6). In contrast, during the later phase, M2 macrophages release anti-inflammatory cytokines such as interleukin-14 (IL-14) and interleukin-3 (IL-3), which promote the repair of myocardial tissue (Ramos et al., 2018). A recent study has demonstrated that M1 macrophages primarily facilitate the germination of blood vessels, while M2 macrophages primarily facilitate the maturation and quiescence of newly formed blood vessels. Prolonged exposure to M1 results in the deterioration of preexisting blood vessels, indicating that timely restoration of inflammatory macrophages following MI/R polarization is crucial for angiogenesis. (Kim et al., 2021, Graney et al., 2020). The phenomenon of macrophage phenotypic switching implies that the M1/M2 transition is governed by a multifaceted array of stimuli. Additional investigation is required to determine the optimal stage of phenotypic switching for implementing interventions that yield the most advantageous outcomes.

### 2.2. Cell death

**Figure 1.**
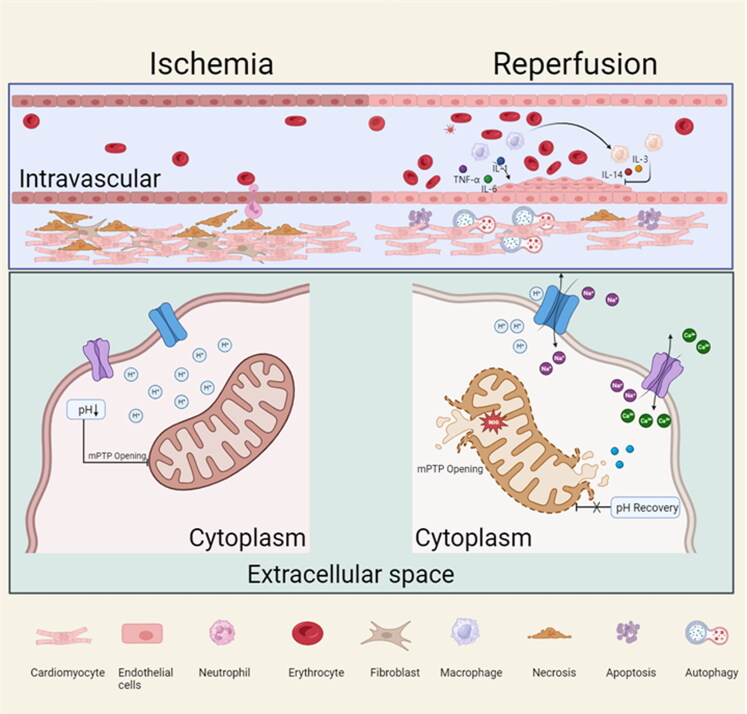
Major pathological mechanisms of MI/RI. Multiple cell death mechanisms including necrosis, apoptosis, autophagy, and massive production of ROS are involved in MI/RI. The opening of the mPTP pore is inhibited in cardiomyocytes under ischemia. Reperfusion is accompanied by a rise in pH and upregulation of Ca^2+^, which prompts the opening of the mPTP and further promotes the production of ROS.

**Figure 2.**
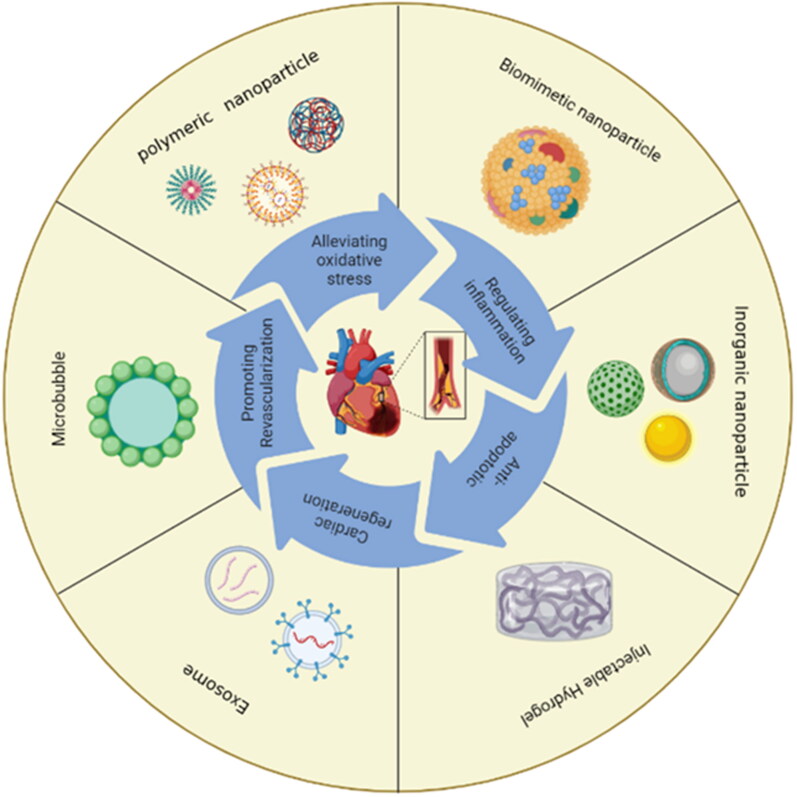
Common nanocarriers for myocardial ischemia-reperfusion therapy.

Extensive and permanent necrosis of cells inside the heart after MI is an important feature. While reperfusion therapy can effectively decrease the size of the infarct, the swift reintroduction of oxygen can result in the generation of excessive ROS, an overload of Ca^2+^, and rapid pH correction. These events can activate multiple pathways of cell death, including apoptosis, autophagy, ferroptosis, necroptosis, and other regulated modes of cell death (Figure 1) (Zhao et al., 2022). The process of cardiomyocyte apoptosis is triggered by both extrinsic factors such as sarcomeric receptors and intrinsic factors involving the release of Cyt c from the impaired mitochondria (Heusch & Gersh, 2017). The opening of mPTP marks the beginning of the endogenous (also known as mitochondrial apoptotic pathway). The mPTP stays blocked when there is an ischemia state. However, the mPTP opens with mitochondrial oxidative stress during the first minutes of significant ROS release and Ca^2+^ overload after cardiac reperfusion. Cyt c is then ejected from the mitochondria and moves to the cytoplasm, where it attaches to Apaf-1 and creates apoptotic bodies, triggering a series of caspase events that eventually lead the cell into apoptosis (Kristen et al., 2013, Marin et al., 2021, Spierings et al., 2005). In the 1990s, the autophagy received widespread attention after Yoshinori Ohsumi studied autophagy genes in yeast cells (Mizushima et al., 2011). By degrading their proteins and organelles in lysosomes, cells may defend themselves through a process called autophagy, which is controlled by a set of genes called autophagy-associated genes (ATGs) (Heusch & Gersh, 2017). The activation of AMP-activated protein kinase (AMPK) is linked to autophagy in the ischemic cardiomyocytes (Matsui et al., 2007). This process has a beneficial and protective impact on the ischemic myocardium by promoting the generation of glycolytic ATP. In contrast, the occurrence of autophagy during the reperfusion phase is associated with an increase in Beclin 1 expression, but not with AMPK activation. Additional research on *beclin 1*± mice indicates that the activated autophagy during the I/R process may have harmful effects (Przyklenk et al., 2012, Matsui et al., 2008, Matsui et al., 2007). Ferroptosis is an iron-dependent controlled cell death and is primarily triggered by excessive Fe^2+^ accumulation and the inactivation of glutathione peroxidase 4 (GPX4). It is characterized by the buildup of lipid hydroperoxides to deadly levels, causing oxidative damage to cell membranes (Heusch & Gersh, 2017). The Fenton reaction, which converts Fe^2+^ to Fe^3+^, can trigger lipid peroxidation and stimulate lipoxygenases to generate substantial quantities of ROS, thereby leading to Ferroptosis. The primary endogenous mechanism for preventing peroxidation is GPX4. The high extracellular concentration of glutamate inhibits the X_c_^-^ system and depletes intracellular cystine. Cystine is converted to cysteine to produce glutathione (GSH), a cofactor of GPX4 (Wu et al., 2021). Heart systolic dysfunction and left ventricular dilation were improved by the apoptosis inhibitor emricasan and the necrosis inhibitor necrostatin-1 in mice during the acute phase (4 hours) of I/R, but not during the prolonged phase (3 or 7 days). However, cardiac systolic dysfunction was prevented when Fer-1, an inhibitor of ferroptosis, was given to patients within a week after their MI/RI. In light of this surprising finding, ferroptosis may be present in late MI/RI (Cai et al., 2023). It is plausible that distinct time intervals after reperfusion may present opportunities to modulate regulated cell death pathways, thereby offering potential targets for pharmacological interventions aimed at protecting cardiac function (Figure 2).

## 3. Nanocarriers for ischemic cardiomyopathy

### 3.1. Polymeric nanoparticles

Polymeric nanoparticles (NPs) are generated through the utilization of polymers, which typically encompass nanocapsules and nanospheres, with a particle size distribution ranging from 1 to 1000 nm. They have become common drug delivery vehicles owing to their biodegradability, capacity for controlled release, and enhanced bioavailability of the encapsulated cargo (Zielińska et al., 2020). For example, polymer nanoparticles constructed by polylactic acid (PLA), glycan acid (PGA) and polylactic acid saccharic acid (PLGA) are often used to carry therapeutic drugs, DNA and proteins, etc. At present, the functional application of these pharmaceutical polymer materials has been approved by the US Food and Drug Administration and the European Medicines Agency (Chenthamara et al., 2019). It is widely acknowledged that the physicochemical characteristics of nanoparticles, including their dimensions, morphology, and surface functionalization, have a significant impact on the biodistribution and uptake of administered nanoparticles. The intravenous administration of Cyclosporine A, which is an inhibitor of mPTP, results in a potent immunosuppressive side effect. Ikeda et al administered intravenous injections of PLGA NPs containing CsA (CsA-NPs) to the myocardial mitochondria of mice with I/R. The concentration of CsA-NPs used was five-fold higher than that of CsA alone, resulting in a reduction of I/R injury that was at least 25-fold more effective. Additionally, the administration of CsA-NPs improved myocardial remodeling in mice after a period of 4 weeks (Ikeda et al., 2016). The enhanced efficacy of nanocarriers in vivo can be attributed to their capacity to prevent rapid degradation of vulnerable molecules and prolong the in vivo circulation time (Yajima et al., 2019). Additionally, the enhanced penetration and retention (EPR) effect, which is characterized by increased permeability of tissue vessels following injury in the ischemia-reperfused heart, has been demonstrated to facilitate passive targeting of NPs. The biodistribution of NPs with core diameters ranging from 20 nm to 2 μm was evaluated in vivo after 30 minutes of administration in mice. The results confirmed that NPs with diameters between 20 and 200 nm are most suitable for passive targeting of the injured left ventricle (Lundy et al., 2016). After exploiting the temporal and spatial fluctuations of MI/RI, combined early-phase inhibition of mPTP opening and late-phase monocyte-mediated inflammatory responses yielded enhanced cardioprotection. (Ikeda et al., 2021). Through the development of micro and nanotechnology, drugs can be delivered through new pathways. Cardiac patches formed by injecting a mixed solution of antioxidant drugs Alpha lipoic acid (LA) and PLGA on an electrostatic spinning device. LA@PLGA (2:8) showed good drug release. In a mouse model, attenuation of deleterious pathologies including oxidative stress, DNA damage, and cytokine-related processes was observed, showing its great potential as a therapeutic approach (Xie et al., 2023).

**Figure 3.**
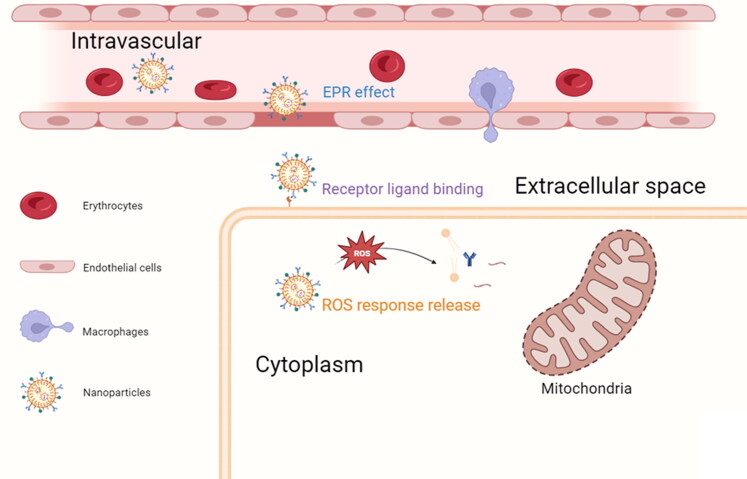
Targeting and controlled release of polymer nanoparticles. Nanoparticles are passively targeted to the site of myocardial injury by means of the EPR effect, followed by ligand-mediated intracellular entry and release of drugs for MI/RI treatment under the stimulation of ROS.

In addition, various innovative ROS-responsive polymeric nanoparticles have been proven for drug administration, which was prompted by the considerable generation of ROS in the cardiac danger zone (Figure 3). By fuzing a dimeric copolymer of PEG and poly(propyleneglycol) (PPS), Li’s team created an amphiphilic molecule that, when exposed to ROS, self-assembles to release the insoluble drug ginsenoside Rg3, which has been shown to diminish myocardial oxidative stress and fibrosis and pave the way for the clinical application of insoluble natural products. Molecular docking and gene silencing experiments confirmed that Rg3 blocked FoxO3 to reduce inflammation and oxidative damage (Li et al., 2020). In an alternative scenario, PLGA NPs functioned as depots for dexamethasone (DXM) that was enclosed within a coating of cRGD-pPEG-modified, ditelluride cross-linked polyethyleneimine (RPPT). nanocomplexes (NCs) that were modified with cRGD were able to effectively target and penetrate inflamed endothelial cells. Within these cells, RPPT was degraded sensitively due to the overproduction of ROS. This degradation then triggered the release of intracellular siVCAM-1, which ultimately enhanced the efficiency of VCAM-1 silencing. The significant reduction of neutrophil infiltration into the ischemic myocardium and the restoration of cardiac function were observed due to the complementary functions of DXM and siVCAM-1, resulting in potent anti-inflammatory efficacy and attenuation of MI/RI. This study presents a viable strategy for the regulated co-administration of small interfering RNAs (siRNAs) and pharmaceutical agents (Hou et al., 2022). Interestingly, dopamine, a clinically used inotropic agent for improving cardiac function in cardiac patients, can itself self-assemble to form polydopamine nanoparticles (PDA NPs). The animal model showed that the 24-h survival rate was 95% in the PDA NPs group and 70% in the myocardial I/R group. Quantitative analysis of Evans blue/TTC staining showed that the average infarct size after surgery was in the I/R group versus the PDA NPs group (36.8% vs 20.2%) (Zhang et al., 2021c). Furthermore, the toxicity of polymer nanoparticles with different particle sizes, shapes, surface modifications, stability in the presence of serum, and other properties need to be further evaluated and determined. It is crucial to set up quality-controlled formulation preparation, quality evaluation, and quantitative characterization methods for the current research status of polymeric NPs (Zielińska et al., 2020).

### 3.2. Inorganic nanoparticles

Inorganic NPs can also be metallic NPs such as magnetic nanoparticles, gold nanoparticles, and nonmetallic nanoparticles including carbon nanoparticles and mesoporous silica nanoparticles, depending on their composition (Zhang et al., 2020, Li et al., 2019).

Iron oxide nanoparticles exhibit superparamagnetic characteristics when their size is below 10–20 nm (Ali et al., 2016). The nanoparticle exhibits a high net magnetic moment solely under the influence of an external magnetic field. The dissipation of the net magnetic moment upon turning off the external magnetic field makes it a promising contrast agent for disease diagnosis and drug delivery (Laurent et al., 2008). Additionally, magnetic nanoparticles (MNPs) have the ability to cause localized hyperthermia in the presence of an external magnetic field, which enables them to target and eliminate cancer cells (Ali et al., 2016, Zhou et al., 2014). They possess a significant surface area to volume ratio, which increases as their size decreases. This characteristic allows for flexible surface modification but also makes them easily identifiable and engulfed by macrophages. To address this issue, hydrophilic polymers like PEG are often used to coat nanoparticles, prolonging their presence and reducing clearance by the reticuloendothelial system (RES) (Zhou et al., 2014). In a study, ferumoxytol, a type of superparamagnetic iron oxide nanoparticles (SPIONs) with a carboxymethyl dextran polymer shell, was utilized as an MRI contrast agent. The SPIONs were attached to VEGF-165, a major isomer with angiogenic effects in MI/RI mouse models, to form a complex. This complex enabled the in vivo distribution of particles to be monitored by MRI, thereby facilitating the assessment of therapeutic efficiency and/or localization of specific cell populations. The vascular endothelial growth factor is crucial for promoting angiogenesis. Moreover, both substances have already received legal and clinical approval, respectively, which significantly reduces the clinical translation time (Bietenbeck et al., 2019). Meanwhile, A ‘vesicle shuttle’ made of nanoparticles was reported by Liu’s team. The complex comprised of a Fe_3_O_4_ core and a silica shell coated with PEG, which binds to two antibody types through hydrazone bonds. Magnetic guidance of CD63-expressing exosomes in infarcted tissue of rabbit and rat models of MI results in decreased infarct size, enhanced left ventricular ejection fraction, and promoted angiogenesis. The core-shell-corona structure of vesicle shuttles enabled the effective collection, transportation, and release of circulating exosomes to specific regions of the organism. (Liu et al., 2020). However, MNPs tend to cause aggregation, and surfactants or polymers are required in the fabrication process to prevent this process (Ali et al., 2016). Iron oxide nanoparticles with magnetic properties induce oxidative stress in the myocardium and worsen cardiac damage resulting from excessive iron accumulation. PEG-coated magnetic iron oxide nanoparticles (PEG-IONPs) exhibit notable accumulation in the kidney and heart even at low doses. Nanoparticle injection resulted in elevated kidney iron levels in rats relative to the control group at the 2-hour and 7-day intervals (Skoczeń et al., 2018).

In addition to metal nanoparticles, mesoporous silica nanoparticles (MSNs) have gained great potential in recent years for biopharmaceutical applications. MSNs are porous materials characterized by stable structure, rich surface chemistry, and high dispersibility (Li et al., 2019). Among them, MSNs have adjustable pore size and flexible drug loading and release characteristics, making them a new star in drug vehicles (Liu et al., 2021, Abu-Dief et al., 2022). In a study by Li et al, MNPs surface modified with CD11b antibody loaded with notoginsenoside R1 ((MSN-NGR1-CD11b) protected myocardium by modulating macrophage phenotype and promoting angiogenesis, attributed to the aggregation of monocytes and neutrophils with high early CD11b expression in MI giving good tropism of the nanoparticles (Li et al., 2022b). With the deepening of gene-drug research, researchers recently have also developed MSNs for simultaneous loading and on-demand release of nucleic acids and chemical drugs. For example, cardiomyocyte-targeted mesoporous silica nanoparticles (MSNs) were developed for ROS-ultrasensitive co-delivery of dexamethasone (Dex) and RAGE small interfering RNA (siRAGE) to attenuate myocardial inflammation. The MSN drug delivery system simultaneously releases Dex and siRAGE in ROS high expressing myocardium, thereby mediating effective silencing of RAGE (72%) and synergistic anti-inflammatory effects (Lan et al., 2022). In contrast to the conventional ROS, pH response, Xia and his group have synthesized a temperature-sensitive polymer poly(N-n-propylacrylamide-co-Ntert-butyl acrylamide) (PNNTBA) modified MSNs (DATS-MSN) delivering hydrogen disulfide (H_2_S), a gas molecule considered to be the third most important endogenous gas molecule in the organism besides nitric oxide (NO) and carbon monoxide (CO), with multiple functions such as antioxidant, anti-inflammatory, angiogenic, and neuromodulatory. The results showed a reduction in the expression of inflammatory factors TNF-α and IL-1β and a decrease in the rate of apoptosis and lactate dehydrogenase activity in I/R hearts (Xia et al., 2022). The nano-formulation is virtually avirulent in vivo and has also become a therapeutic vehicle for many drug candidates.

### 3.3. Microbubbles

**Figure 4.**
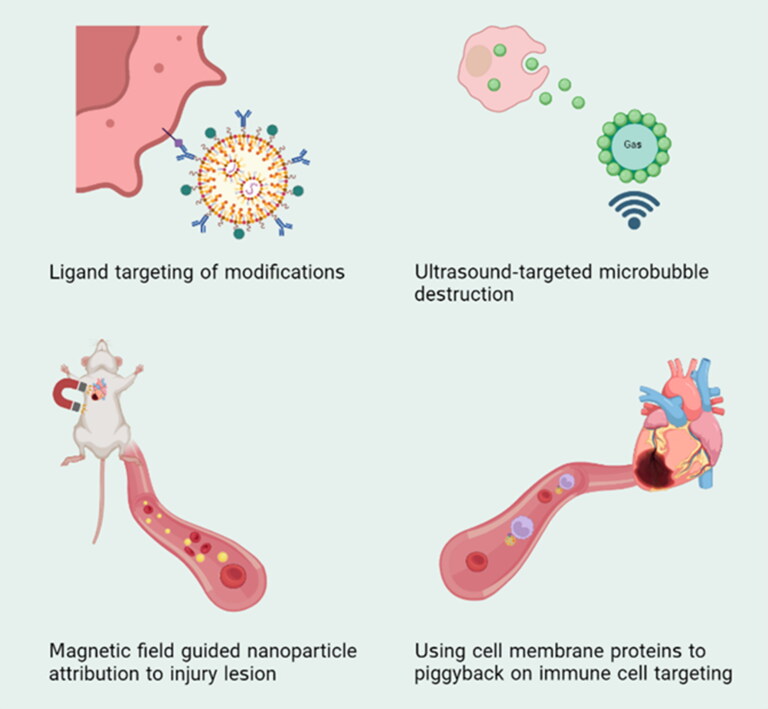
Commonly used targeted myocardial drug delivery strategies.

Microbubbles consist of an inner core of gas with low solubility in water and a shell of a macromolecular material that exhibits an acoustic impedance mismatch between biological fluids and tissues making them suitable for use in ultrasound imaging and as potential cardiovascular drug delivery vehicles (Unger et al., 2014). A potential therapeutic approach for the targeted delivery of stem cells, ultrasound-targeted microbubble destruction (UTMD) uses the cavitation effect of microbubbles (Figure 4) (Lum et al., 2006, van Wamel et al., 2006, Zhong et al., 2012). Stromal cell-derived factor (SDF-1α) is one of the most important chemokines in the homing of stem cells to the infarcted myocardium. However, spontaneous secretion of SDF-1α is deficient and transient. Su et al. loaded an adenovirus carrying the SDF-1α gene in a microbubble vector and released it by UTMD in AMI rats and observed a significant increase in the number of endogenous bone marrow mesenchymal stem cells (BMSCs) at myocardial infarction sites (Su et al., 2018). Survival of transplanted exogenous stem cells under ischemic conditions is challenging. Sun et al. prepared microbubble preparations from lentiviral PHD2 shRNA transduction of BMSCs with increased BMSC survival, reduced cardiomyocyte apoptosis, reduced infarct size, increased vascular density, and improved cardiac function in rats compared with controls, providing a good strategy to improve the effectiveness of stem cell therapy after AMI (Sun et al., 2020).

Gene drugs, such as plasmid DNA and siRNA, may be very effective when used at low doses on a limited loading of microbubbles (Unger et al., 2014). Cationic microbubbles (CMBs) have strong DNA binding ability due to a positive charge and make negatively charged DNA not degrade in blood, to improve gene transfection ability (Sun et al., 2013, Nomikou et al., 2012). Yu et al. developed UTMD combining with the human SDF-1α- nuclear factor κB plasmid (phSDF-1α-NFκB) to enhance the stromal cell-derived factor 1 alpha (SDF-1α) gene transfection rate. It suggested that the increased expression of the SDF-1α gene can significantly improve cardiac function, resulting in large myocardial perfusion and more angiogenesis (Yu et al., 2022). Also in large mammalian dogs UTMD -mediated Ang 1 gene transfection could be observed from day 1 to 1 month after MI, the UTMD-Ang1 group showed a significant reduction in plasma norepinephrine and N-terminal pre-B-adrenergic peptide (NT-proBNP) from day 1 to 1 month after MI, exhibiting significant effects of promoting angiogenesis and reversing LV structure and sympathetic remodeling (Cao et al., 2021). UTMD has the advantages of low toxicity, low invasiveness, high specificity, and low immunogenicity, which make it widely used. However, it still has many shortcomings, which need our attention and improvement. First of all, there may be adverse reactions such as capillary rupture and inflammatory cell infiltration during ultrasound irradiation. and these safety issues need to be urgently addressed. Secondly, the parameters can affect the transfection efficiency, but the optimal ultrasound parameters have not been determined (Qian et al., 2018).

### 3.4. Exosomes

In the realm of secretory membrane vesicles, exosomes occupy a unique niche. Exosomes, vesicles with a size range of 50-150 nm generated by the twofold invagination of the plasma membrane, transport macromolecules including proteins, nucleic acids, and lipids between cells and play a crucial role in this process. (Chen et al., 2017). Exosomes possess innate material transport capabilities, inherent long-term recycling capacity, and favorable biocompatibility, among other attributes, rendering them highly promising as drug delivery vehicles (Liu & Su, 2019). In general, exosomes are derived from different cells and the heterogeneity of their size and content can reflect their state and type of origin, which makes exosomes not only promising diagnostic biomarkers for diseases but also the abundance and complexity of the contents involved in transport (Xiong et al., 2021). Low cell homing effectiveness in the infarcted myocardial has been one of the main difficulties of cell treatment after AMI, in addition to poor survival, low proliferation rates, engraftment, and insufficient differentiation of the transplanted cells (Ziegler et al., 2017). Additionally, investigations have revealed that the exosomes discharged by MSCs exhibit properties such as anti-apoptotic, vascular regenerative, and inflammatory modulation (Figure 4) (Xiong et al., 2021, Zhao et al., 2019). Zhao et al. observed that exosomes could effectively convert macrophages to M2 type by intra-myocardial injection of MSC-derived exosomes (MSC-exo) in mice, thereby reducing the inflammatory storm and promoting subsequent repair with reduced infarct size. Further analysis and validation showed that mi-182 in exosomes is involved in polarization by functioning in the TLR4/NF-κB/PI3K/Akt signaling cascade (Zhao et al., 2019). Wang et al. evaluated the therapeutic effects of MSCs from the endometrium, bone marrow, and adipose tissue in the myocardium of infarcted rats, respectively, and found that endometrial MSC-derived exosomes had the best efficacy, which could be explained by paracrine enhancement of miR-21 expression, which has critical effects on downstream cell survival and angiogenesis (Wang et al., 2017). Recent research has revealed that the circadian rhythms of macrophages, regulated by miR-21, have a notable impact on the vulnerability of plaque lesion growth and rupture. It was further observed that prophylactic measures aimed at controlling miR-21 expression were effective in reducing susceptibility to early morning plaque rupture (Schober et al., 2021). Furthermore, exosomes derived from hypoxic bone marrow-derived mesenchymal stem cells (BMSCs) containing miR-98-5p have been found to inhibit myocardial enzyme levels, oxidative stress, inflammatory response, macrophage infiltration, and infarct size in I/R myocardial tissue. This is achieved through the inhibition of toll-like receptor 4 (TLR4) and activation of phosphoinositide 3-kinase/protein kinase B (PI3K/Akt) signaling pathways in rats with MI/RI (Zhang et al., 2021a). Shen et al. also further studied that MSC-derived exosomes promote their differentiation to M2 phenotype through miR-21-5P and reduce the expression level of inflammatory factors (Shen & He, 2021). Exosomes are highly heterogeneous, and changes in donor cells during the processing and modification of engineered exosomes may affect the content or protein composition of exosomes, making it difficult to achieve therapeutic standardization (Han et al., 2022). The emergence of precision medicine has led to the utilization of exosomes for the screening of single-component microRNA. This approach offers the advantage of increased precision and uniqueness. Studies involving the administration of modified oligonucleotides (agomir) in mouse I/R myocardium, as well as MSCs or MSC-exos, have demonstrated that miR-125a-5p agomir regulates macrophage, fibroblast, and cardiomyocyte functions. Furthermore, this approach has been shown to improve cardiac performance and remodeling in myocardial I/R pigs without an increase in arrhythmia frequency or liver, kidney, or cardiac toxicity (Gao et al., 2023).

However, targeting the receptor cells remains a problem. Using molecular cloning and lentiviral packaging techniques, Wang et al. fused exosome-enriched membrane proteins with ischemic myocardial targeting peptide (IMTP) and were able to be effectively internalized by hypoxia-injured H9c2 cells and had more accumulation in mouse ischemic myocardium compared to blank exosomes (Wang et al., 2018). Animal experiments have shown that the biodistribution of exosomes is mainly concentrated in the liver, lung, kidney, and spleen, regardless of the origin or size of the exosomes (Kang et al., 2021). Zhang et al. recently incorporated dendritic cell-derived exosomes (DEXs) with alginate hydrogel (DEXs-Gel) and applied them to MI model mice. The result showed that it had better effects on immune regulation, anti-apoptosis, and angiogenesis in myocardial repair after MI due to the prolonged retention time of DEXs (Zhang et al., 2021b).

### 3.5. Membrane-camouflaged nanoparticles

The first report of nanomaterials encapsulated by erythrocyte membranes in 2011 (Hu et al., 2011), a series of studies on the use of cell membrane surface ligands to promote targeted delivery of nanoparticles have achieved promising results. Nonetheless, the complexity of MI/RI pathomechanisms involving many membrane proteins associated with vascular injury and susceptibility to infarction poses a challenge to the full scale-up of membrane protein mimetic nanomedicines, which is currently being addressed through the implementation of top-down nanoparticle masking on natural biological membranes (Oroojalian et al., 2021). Platelets, immune cell membranes, tumor cell membranes, and even organelle membranes have joined the ranks of bionic materials one after another in the last decade (Qiu et al., 2019, Zhou et al., 2022, Pitchaimani et al., 2018). The therapeutic efficacy of platelet (PLT)-mimetic MI/RI is noteworthy due to the rise in circulating monocyte-platelet aggregates observed in individuals with acute coronary syndromes. By virtue of the physiological characteristics of platelet membrane carrying monocyte ‘hitch’ to the homing of ischemic myocardium, Bill et al. constructed platelet-like proteoliposomes (PLPs), biomimicking platelet interactions with circulating monocytes, PLPs do not aggregate on uninjured endothelium but do accumulate at ischemic myocardial sites 72 h after infarction (Cheng et al., 2016). Utilizing ultrasonic or mechanical extrusion methods to combine cell membranes with nano-formulations, allows proteins on the biofilm, especially P-selectin, to be modified to the outer layer of the formulation and bind to the P-selectin glycoprotein ligand-1 (PSGL-1) to achieve active targeting (Figure 4) (Lu et al., 2019). It is noteworthy that while cationic nanoparticles have demonstrated efficacy in gene drug delivery, an excess of positive charge in cations can outcome in binding to the cell surface, leading to cell membrane damage and potential cytotoxicity (Ho et al., 2019). The implementation of a bionic membrane as a protective covering can effectively prevent this effect. Furthermore, the fusion between cationic nanoparticles and cell membranes can be attributed to their mutual attraction. The delivery efficacy of hybrid membranes was demonstrated through the incubation-extrusion of cationic liposomes with platelet membranes that were encapsulated with miR-21-loaded MSNs. platelet membrane proteins exhibited a predilection for Ly6C^high^ monocytes, thereby facilitating the effective dissemination of miR-21 to acceptor cells, culminating in the M1/M2 reprogramming of macrophages (Tan et al., 2021). Due to the discrepancy between the regulation of macrophage reprogramming that occurs two days or more after IR and the EPR effect impact that is shown at the site of cardiac damage 24 hours after IR, active targeting is an important factor to take into account (Nguyen et al., 2015). Similar to this, the chimerization of ROS-sensitively characterized diselenide bond-modified liposomes with platelet membranes achieved localized ROS-responsive release of RvD1. It significantly improved myocardial function in the MI/RI mouse model, which showed the highest LVEF preservation in the PLP-RvD1 group compared to the other groups (43.85 ± 1.57% vs. 29.09 ± 1.32%, PLP-RvD1 vs. Ctrl) (Weng et al., 2022). In addition, the novel nanoformulation CsA@PPTK exhibits superior targeting of CsA in ischemic myocardium as compared to its predecessors (Ikeda et al., 2016). This is achieved through the wrapping of platelet membranes around CsA in ischemic myocardial tissue, resulting in reduced accumulation of CsA@PPTK in the liver. Furthermore, in the high reactive oxygen species (ROS) environment of ischemic myocardium, CsA is encapsulated and subsequently released in the ROS-responsive material PTK. The synergistic effect produced by CsA in the treatment of MI/RI is attributed to this mechanism (Li et al., 2022a). Similarly, platelet membrane fusion with other nanocarriers such as extracellular vesicles, exosomes, and polymeric NPs for delivery of drug formulations can also actively target the damaged heart, and validation in animal models has shown great potential for the treatment of ischemic diseases (Li et al., 2021, Hu et al., 2021, Wang et al., 2022a). On the one hand, platelets are easily extracted from blood and have greater potential for clinical translation. At the same time, platelet membrane surfaces packed with the transmembrane proteins GPIV, GPV, GPVI, GPIX, and CLEC-25, the immunomodulatory proteins CD47, CD55, and CD59, as well as several integral proteins, also offer suggestions for the creation of new targets for the treatment of other diseases. Among them, CD47 can protect the body from recognition and attack by disguising itself and releasing the ‘don’t eat me’ signal (Zargar et al., 2019). This novel therapeutic modality using platelet membranes for drug delivery has great therapeutic potential in vascular inflammatory diseases (Hu et al., 2016, Hu et al., 2015). Macrophage and neutrophil membranes can be fused with nanoparticles for the purpose of participating in myocardial ischemia-reperfusion therapy (Chen et al., 2022, Wei et al., 2021). The diverse surface area properties and fusion methods of nanoparticles result in varying fusion efficiencies, making it challenging to achieve uniform characterization. Additionally, successful fusion should ensure the complete expression of membrane proteins on the surface to achieve the desired targeting effect (Oroojalian et al., 2021).

### 3.6. Injectable hydrogels

**Figure 5.**
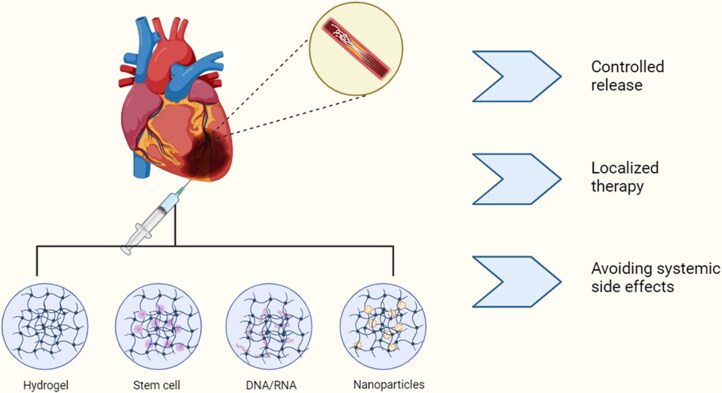
Injectable hydrogels and their applications.

Hydrogels have an insoluble three-dimensional polymeric retinoid structure and are characterized by high biocompatibility, biodegradability, and high absorption, and are thus used in the in vivo delivery of drugs, nucleic acids, and cells. (Shi et al., 2022) The concept of injectable biomaterials for the treatment of MI was first introduced in the early 2000s (Christman et al., 2004). The injectable hydrogel can form a meshwork structure at a certain temperature, providing a morphological environment for cardiomyocytes to support and trap growth factors and promote myocardial repair. In addition, these hydrogels could exert local therapeutic effects in the injected portion and release the drug in a controlled manner according to the pathological environment, reducing the adverse effects associated with systemic administration (Figure 5). Collagen, gelatin, laminin, chitosan, hyaluronic acid, and other natural biomaterials with low toxicity and low immunogenicity have been widely used in cardiac tissue engineering (Alagarsamy et al., 2019). In this context, a variety of readily degradable bioactive molecules in vivo, such as growth factors, functional peptides, and stem cells, are widely used to promote myocardial regeneration. The 7Ap peptide is encoded by the short open reading frame (sORF) of the HDAC7 gene, whose phosphorylation promotes in situ tissue repair by mobilizing and recruiting endogenous stem cell antigen-1-positive (Sca-l^+^) stem cells. Zhang et al. recently found that loading collagen hydrogels with 7-amino acid peptide (7Ap) enhanced H9c2 cell survival in vitro and recruitment and differentiation of stem cell antigen-1-positive (Sca-1^+^) stem cells in vivo and angiogenesis to promote myocardial cell cycle progression, suggesting that 7-Ap collagen could be a candidate for myocardial repair (Zhang et al., 2019). VentriGel, an extracellular matrix (ECM) hydrogel biomaterial derived from porcine myocardium, recently had a preliminary reliable safety and feasibility in 15 patients with post-myocardial infarction left ventricular dysfunction in the first clinical trial evaluating transendocardial injectable biomaterials (NCT02305602) (Traverse et al., 2019). At the same time, synthetic hydrogels are receiving increasing attention because of their availability, low manufacturing costs, robust mechanical properties, and functional control by physical or chemical methods (Liao et al., 2020). For example, Zhou’s team prepared conductive hydrogels by combining graphene oxide (GO) nanomaterials with oligomeric (polyethylene glycol) fumarate (OPF) hydrogels with good electrical conductivity and mechanical properties to activate the classical Wnt signaling pathway to form electrical connections and mechanical support between scarred and healthy myocardium. wall thickness was significantly preserved (from 0.37 ± 0.096 mm to 0.77 ± 0.079 mm) and the infarct size was significantly reduced (from 50.7% to 31.7%) (Zhou et al., 2018). In addition to mechanical support, hydrogels can address the problem of low retention rates in stem cell delivery. lyu et al. integrated prepared human VE-cad-Fc fusion protein-modified particles with human mesenchymal stem cells (hMSC) for injectable hyaluronic acid (HA) hydrogel delivery to confirm the reconstruction and improvement of cardiac function after infarction (Lyu et al., 2020). At the same time, the implantation of stem cells posed some problems. Tachyarrhythmias occurred between the time of implantation of human embryonic stem cell-derived cardiomyocytes into the infarcted pig heart and the recovery of sinus rhythm 4 weeks later (Romagnuolo et al., 2019). Therefore its biocompatibility, degradability, biosafety and low adhesion to cells are issues worth discussing (Liao et al., 2020). Moreover, most hydrogels are administered by intracardiac injection, and the practical handling is more challenging (Table 2).

**Table 2.** Advantages and disadvantages of various drug delivery systems.

Type of drug delivery	Advantages	Disadvantages	References
Polymeric Nanoparticle	Controlled release capability (sustained-release, controlled-release, long-acting), high-compliance drugs, excellent biodegradability, surface modification and targeted easily	Easily trapped by the liver and spleen, part of the nanoparticles easily aggregation and toxicity	(Zielińska et al., 2020, Mitchell et al., 2021)
Inorganic Nanoparticles	Large surface area to volume ratio, great response to electromagnetism, flexible variations in size, structure and geometry	Poor biocompatibility, causing aggregation and inducing oxidative stress, accumulation in the kidneys and heart	(Zhou et al., 2014, Ali et al., 2016, Li et al., 2019, Mitchell et al., 2021)
Microbubbles	In response to ultrasound to target and release the drugs, strong DNA binding ability	Capillary rupture and inflammatory cell infiltration are difficult to determine ultrasound parameters. immature techniques for preparing and modifying microbubbles	(Qian et al., 2018)
Exosomes	Innate material transport capabilities, inherent long-term recycling capacity, favorable biocompatibility	Difficult to target receptor cells, easily absorbed by the lungs and liver, with complicated contents, difficult to ensure uniform quality	(Liu & Su, 2019, Xiong et al., 2021, Wang et al., 2018, Smyth et al., 2015)
Membrane camouflaged nanoparticles	Excellent natural targeting, compatible with a variety of engineered nanoparticles, against the phagocytosis of liver and spleen	Differential fusion efficiency and immunoreactivity of surface membrane proteins	(Oroojalian et al., 2021, Zargar et al., 2019)
Injectable hydrogels	Controlled release, local treatment of myocardial tissue, loaded with multiple drugs (nanoparticles, drugs, DNA, etc.)	Complex mode of administration (usually intramyocardial injection), relatively poor security	(Liao et al., 2020, Shi et al., 2022)

## 4. Conclusions and prospects

In recent years, based on the booming development of DDSs, especially nanocarrier systems, their application in cardiovascular diseases has also received increasing attention from researchers. Most of the drug carriers are still in the laboratory research stage and few have entered clinical studies, and they face many technical challenges such as biocompatibility, controllability of technical manipulation and effectiveness of targeted delivery (Guan et al., 2021). This suggests that there is still a great deal of research to be done on drug carriers for future cardiovascular applications.

Reperfusion attenuates myocardial ischemia and hypoxia to a large extent, but the ensuing increase in pH generate, excessive ROS and Ca^2+^ overload, which leads to dysregulated oxidative stress and massive apoptosis or necrosis in cardiomyocytes (Wu et al., 2018, Bugger & Pfeil, 2020). Several approaches are available to improve myocardial function by utilizing the responsiveness of external conditions (magnetism, ultrasound, etc.) and the internal microenvironment (ROS, etc.) to enhance drug colonization and release in sites of myocardial injury (Zhao et al., 2022, Liu et al., 2020, Sun et al., 2020). In spite of this, the expected efficacy of current therapeutic drugs for MI/RI is not obvious, and the possible reasons include hepatic and splenic sequestration, lack of myocardial targeting of the drug, and low accumulation of the drug under the rapid blood flow impact, which inevitably requires high-dose administration and is likely to lead to severe systemic adverse effects. The passive accumulation of the nano-drug formulation into the myocardium using the EPR effect of the nano-formulation at the site of the ischemic myocardium, combined with the receptors expressed at the site of myocardial injury, further enhances the accumulation of the drug. Especially for some highly effective drugs with poor targeting, poor water solubility, easy degradation and short half-life in vivo, the development of novel drug delivery systems to deliver these drugs efficiently to the effector part can largely improve the therapeutic effect of the drugs, which will have high research value in the future. Meanwhile, gene and stem cell therapies, which can solve a series of problems starting from the mechanism of myocardial ischemia, are becoming a hot research topic. Despite the problems of easy degradation, low transfection efficiency and low transplantation rate, gene and stem cell therapies are likely to become a new type of therapeutic means in the future with the continuous improvement of drug delivery system. In addition, there is much research exploring how to integrate the advantages of each vector so that they complement each other. In particular, the use of cell membranes, and especially the tendency of inflammatory cells to injured myocardial sites, can be mimicked to a degree that is difficult for engineering to achieve by utilizing the interactions of multiple natural surface ligands on their membrane surfaces. On the other hand, there is a crossover process between post-infarction myocardial inflammation and fibrosis, and by utilizing the optimal therapeutic window after myocardial infarction, drugs can be effectively delivered to the myocardial site at the optimal time to modulate inflammatory cells, especially macrophage M1/M2, which can achieve effective improvement of myocardial function and fibrosis.

However, most studies are still at the rodent and a small number of non-human mammalian levels and the animal models do not fully simulate the human pathological state, mostly using mice that have surgically reached the ischemic state rather than the chronic model, and mostly using young mice as experimental subjects, which is also not consistent with the age of onset of MI/RI. Meanwhile, the biocompatibility and safety of various emerging synthetic materials also need to be further evaluated and optimized. The particle size, morphology, electrical potential, and surface modification of nanoparticles all affect their distribution in organisms. In particular, the accumulation in the liver and kidney needs attention, for which a standardized assessment system is essential. In addition, the fundamental challenges of achieving monodispersity, controlling shape and size, and reproducibility in industrial manufacturing also need to be faced.

In summary, nano-delivery systems need to target the pathological aspects of myocardial cell death, oxidative stress, inflammation and fibrosis to achieve optimal therapeutic effects. These drug delivery systems require not only excellent targeting, release of the right drug dose at the right time, but also a good biosafety. Therefore, the development of biomimetic nanoformulations for myocardial drug delivery systems has a long way to go. In conclusion, the future development of low-toxicity and efficient DDSs for clinical cardiovascular diseases will have a promising prospect.

## Data Availability

Data availability is not applicable to this article as no new data were created or analyzed in this study.
